# microRNA-96 promotes occurrence and progression of colorectal cancer *via* regulation of the AMPKα2-FTO-m6A/MYC axis

**DOI:** 10.1186/s13046-020-01731-7

**Published:** 2020-11-12

**Authors:** Caifeng Yue, Jierong Chen, Ziyue Li, Laisheng Li, Jugao Chen, Yunmiao Guo

**Affiliations:** 1 Department of Laboratory Medicine, Central People’s Hospital of Zhanjiang, Guangdong Medical University Zhanjiang Central Hospital, 236 Yuanzhu Road, 524045 Zhanjiang, P. R. China; 2 Division of Laboratory Medicine, Guangdong Provincial People’s Hospital, Guangdong Academy of Medical Sciences, 510080 Guangzhou, P. R. China; 3 Guangzhou Women and Children’s Medical Center, Guangzhou Medical University, 510623 Guangzhou, P. R. China; 4 Department of Laboratory Medicine, The First Affiliated Hospital of Sun Yat-Sen University, 510080 Guangzhou, P. R. China; 5 Department of Oncology, Shenzhen People’s Hospital, Second Clinical Medical College of Jinan University, First Affiliated Hospital of Southern University of Science and Technology, No. 3046, Shennan East Road, Luohu District, 518020 Shenzhen, Guangdong Province P. R. China; 6 Clinical Research Institute of Zhanjiang, Central People’s Hospital of Zhanjiang, Guangdong Medical University Zhanjiang Central Hospital, 236 Yuanzhu Road, 524045 Zhanjiang, Guangdong Province P. R. China

**Keywords:** Colorectal cancer, microRNA-96, m6A modification, AMPKα2, FTO, MYC

## Abstract

**Background:**

Colorectal cancer (CRC) is one of the frequently occurred malignancies in the world. To date, several onco-microRNAs (miRNAs or miRs), including miR-96, have been identified in the pathogenesis of CRC. In the present study, we aimed to corroborate the oncogenic effect of miR-96 on CRC and to identify the specific mechanisms related to AMPKα2/FTO/m6A/MYC.

**Methods:**

RT-qPCR and Western blot analysis were performed to examine the expression pattern of miR-96, AMPKα2, FTO and MYC in the clinical CRC tissues and cells. The relationship between miR-96 and AMPKα2 was then predicted using *in silico* analysis and identified by dual-luciferase reporter assay. Gain- or loss-of-function approaches were manipulated to evaluate the modulatory effects of miR-96, AMPKα2, FTO and MYC on cell growth, cycle progression and apoptosis. The mechanism of FTO-mediated m6A modification of MYC was analyzed *via* Me-RIP and PAR-CLIP analysis. The mediatory effects of miR-96 antagomir on cancerogenesis were validated *in vivo*.

**Results:**

miR-96, FTO and MYC were upregulated, while AMPKα2 was downregulated in CRC tissues and cells. miR-96 could down-regulate AMPKα2, which led to increased expression of FTO and subsequent upregulated expression of MYC *via* blocking its m6A modification. This mechanism was involved in the pro-proliferative and anti-apoptotic roles of miR-96 in CRC cells. Besides, down-regulation of miR-96 exerted inhibitory effect on tumor growth *in vivo*.

**Conclusions:**

Taken together, miR-96 antagomir could potentially retard the cancerogenesis in CRC *via* AMPKα2-dependent inhibition of FTO and blocking FTO-mediated m6A modification of MYC, highlighting novel mechanisms associated with colorectal cancerogenesis.

## Background

Colorectal cancer (CRC) represents the third most frequently occurring cancers on a global scale [1]. CRC is reportedly heterogeneous accompanied by substantial genetic and phenotypic differences between individuals [2]. Approximately 90% of CRC cases are affected by adverse events after pharmacological therapy, and the implementation of pharmacogenomics is currently addressed in enhancing drug safety [3]. Moreover, a great deal of ongoing investigations for identifying molecules involved in the pathophysiology of CRC individualizes therapeutic options in the near future [4–6].

Of note, dysregulation of microRNAs (miRNAs or miRs) has been associated with a wide array of pathological processes due to their abilities to bind to protein-coding mRNAs [7]. There is a paucity of data highlighting the orchestration of miRNAs on initiation, aggressiveness and metastatic potential of malignancies [8–10]. Interestingly, miR-96 has been widely implicated in the progression and metastatic potential of a plethora of cancers, including cervical [11], and ovarian [12] cancers. Although miR-96 is witnessed to link to tumor invasion in addition to cancer cell migration and invasion in CRC [7, 13], little is well-understood regarding its downstream mechanisms related to the pathophysiological events of CRC.

N6-methyladenosine (m6A) modification has been demonstrated to epitranscriptionally control mammalian gene expression in the multiple biological processes; hence m6A and its mediators are expected to be promising therapeutic targets for human cancers [14]. Fat mass and obesity-associated (FTO) gene is a well-known m6A eraser that is upregulated in CRC and reported to interact with c-myc proto-oncogene (MYC) to accelerate CRC cell proliferating and migrating capabilities [15]. AMP-activated protein kinase-alpha2 (AMPKα2), also named PRKAA2, has been stated to be in an inverse correlation with FTO [16].

Here, we addressed the contribution of miR-96 to CRC progression and demonstrated that miR-96 directly targeted AMPKα2 which inhibits the expression of m6A demethylase FTO. More specifically, FTO can activate MYC by reducing the m6A modification of MYC, whereby leading to cancerogenesis.

## Materials and Methods

### Clinical sample collection

Sixty patients (42 males and 18 females, aged 35–75 years, with a mean age of 56.70 ± 9.96 years) diagnosed with CRC at The First Affiliated Hospital of Sun Yat-Sen University from August 2017 to August 2019 were enrolled. None of these patients received radiotherapy or chemotherapy prior to operation, and their tumor and paracancerous tissues were preserved in liquid nitrogen for follow-up studies.

### Microarray-based gene expression profiling

The key miRNA associated to CRC were determined from the existing literature, and expression of key miRNA was determined based on the microarray dataset GSE38389 retrieved from the GEO database. The differentially expressed genes in CRC were obtained by analyzing TCGA database through GEPIA with the threshold as |logFC > 1|, *p* < 0.01. Next, the downstream genes of miRNA were predicted by the databases TargetScan and miRWalk (accessibility < 0.01, au ≥ 0.75). The Venn diagram of the differentially expressed genes and miRNA downstream genes was then plotted. PPI network of the intersected genes was constructed using String, and the PPI network image was drawn using Cytoscape, with the core degree calculated. The genes related to the key genes were predicted by MEM (the first 10,000 with significant co-expression relationship, and 6711 genes left after the removal of duplicates) (https://biit.cs.ut.ee/mem/index.cgi) and LinkedOmics (the first 2000 with significant correlation degree) (http://www.linkedomics.org), and subsequently among which the m6A RNA modification-related genes screened. The downstream genes of m6A RNA modification were predicted from the existing literature, and the expression correlation graph was plotted by GEPIA for verification. TCG Aportal (http://tumorsurvival.org) was used to analyze the relationship between its expression and CRC survival.

### Cell culture and transfection

CRC cells SW480 (ZQ0063), SW620 (LZQ0014), HCT-8 (ZQ0331) (Shanghai Zhong Qiao Xin Zhou Biotechnology Co., Ltd., Shanghai, China) and normal intestinal epithelial cells HIEC (American Type Culture Collection [ATCC], Rockville, MD) were cultured in Dulbecco’s modified Eagle’s medium (DMEM) containing 10% fetal bovine serum (FBS) in a 5% CO_2_ incubator at 37℃. After adherent growth, cells were detached with 0.25% trypsin (Hyclone, South Logan, UT).

Cells were then transfected with the following sequences: negative control (NC) mimic, miR-96 mimic, NC overexpression plasmid (oe-NC), AMPKα2 overexpression plasmid (oe-AMPKα2), FTO overexpression plasmid (oe-FTO), siRNA targeting AMPKα2 (si-AMPKα2), NC shRNA (sh-NC), shRNA targeting FTO (sh-FTO), MYC overexpression plasmid (oe-MYC) singly or in combination. The plasmid pCMV6-AC-GFP for gene overexpression and plasmid pGPU6/Neo for gene silencing were purchased from Fenghui Biotechnology Co., Ltd. (FH1215, Hunan, China) and Shanghai GenePharma Co, Ltd. (Shanghai, China) respectively. The cell transfection was performed with the use of Lipofectamine 2000 reagents (Invitrogen, Carlsbad, CA) (11,668,019, Thermo Fisher Scientific). 4 µg target plasmids and 10 µL Lipofectamine 2000 were diluted in 250 µL Opti-MEM (Gibco) respectively, and then mixed gently. After being allowed to stand for 20 min, the mixture was cultured with the cells in a 5% CO_2_ incubator at 37℃. After 6 h, the medium was replaced with a complete medium for another 48 h culture.

### Reverse transcription quantitative polymerase chain reaction (RT-qPCR)

Total RNA was extracted from tissues or cells by TRIzol reagents (Invitrogen, Carlsbad, CA), and the concentration and purity of the extracted total RNA were detected by a NanoDrop2000 UV microspectrophotometer (1011U, NanoDrop Technologies, Rockland, DE). According to the instructions of TaqMan MicroRNA Assays Reverse Transcription primer (4,427,975, Applied Biosystems, Foster City, CA)/PrimeScript RT reagent Kit (RR047A, Takara, Japan), the RNA was reverse transcribed into cDNA, and primers for miR-96, AMPKα2, FTO, and MYC were designed and synthesized by Takara (Table 1). RT-qPCR was performed on an ABI 7500 instrument (7500, Applied Biosystems, Foster City, CA). The fold changes were calculated using relative quantification (2^−△△CT^ method) normalized to glyceraldehyde-3-phosphate dehydrogenase (GAPDH) or U6.

**Table 1:** Primer sequences for RT-qPCR

Gene	Primer sequence
miR-96	F: 5’-TTGGGTGAAATATATTGTGCGTCTC-3’
	R: 5’-AGCCGAAGTGAGCCACTGAA-3’
U6	F: 5’-GCACCGTCAAGGCTGAGAAC-3’
	R: 5’-AGCCGAAGTGAGCCACTGAA-3’
AMPKα2	F: 5’-GGGACCTGAAACCAGAGAACG-3’
	R: 5’-ACAGAGGAGGGCATAGAGGATG-3’
FTO	F: 5’-TGAAGGTAGCGTGGGACATAGA-3’
	R: 5’-GGTGAAAAGCCAGCCAGAAC-3’
MYC	F: 5’-TTCGGGTAGTGGAAAACCAG-3’
	R: 5’-AGTAGAAATACGGCTGCACC-3’
MYC-m6A	F: 5’-GCATACATCCTGTCCGTCCA-3’
	R: 5’-GTCGTTTCCGCAACAAGTCCC-3’
GAPDH	F: 5’-GCACCGTCAAGGCTGAGAAC-3’
	R: 5’-TGGTGAAGACGCCAGTGGA-3’

Note: RT-qPCR, reverse transcription quantitative polymerase chain reaction; F, forward, R, reverse; miR-96, microRNA-96; m6A, N6-methyladenosine; AMPKα2, AMP-activated protein kinase-alpha2; FTO, fat mass and obesity-associated; MYC, c-myc proto-oncogene; GAPDH, glyceraldehyde-3-phosphate dehydrogenase

### Western blot analysis

The total protein was extracted from tissues or cells using radioimmunoprecipitation assay (RIPA) lysis buffer (P0013C, Beyotime, Shanghai, China) containing phenylmethylsulphonyl fluoride (PMSF). The cell lysate was centrifugated to harvest supernatant. Next, 50 µg protein was subjected to sodium dodecyl sulfate polyacrylamide gel electrophoresis and transferred to polyvinylidene fluoride membranes by a wet transfer method. The membrane was blocked using 5% skim milk powder at indoor temperature for 1 h and then reacted overnight at 4℃ with the following diluted primary rabbit antibodies (Abcam, Cambridge, UK): AMPKα2 (1:500, ab3760), FTO (1:1500, ab126605), and MYC (1:1000, Ab32072) and CDK2 (1:1000, ab32147), CDK4 (1:500, ab137675), Ki-67 (1:1000, ab16667), PCNA (1:1000, ab18197), Bax (1:1000, Ab199677), and Bcl-2 (1:500, ab59348). The next day, the membrane was washed with Tris-buffered saline Tween-20 (TBST), and re-probed with secondary goat anti-rabbit IgG (H + L) horseradish peroxidase (HRP) (ab97051, 1:2000, Abcam, Cambridge, UK) for 1 h. The immunoreactive bands were visualized using enhanced chemiluminescence reagent (BB-3501, Amersham, UK) and proteins were quantified (normalized to β-actin) using a Bio-Rad image analysis system (Bio-Rad Laboratories, Hercules, CA) and Quantity One v4.6.2 software.

### Dual-luciferase reporter assay

Human embryo kidney (HEK) 293T cells were cultured in DMEM containing 10% FBS under 5% CO_2_ at 37℃. The cDNA fragment of AMPKα2-mutant (Mut) containing miR-96 binding site was inserted into the pmirGLO vector. cDNA fragment of AMPKα2-Mut was synthesized by site-directed mutagenesis and then inserted into pmirGLO vector. The inserted sequence was verified to be correct by sequencing (all the above operations were completed by RIBOBIO, Guangzhou, China). HEK293T cells underwent co-transfection with pmirGLO-AMPKα2-wild type (Wt) or pmirGLO-AMPKα2-Mut recombinant vector and miR-96 mimic or NC mimic for 48 h. The activity of renilla luciferase and firefly luciferase was determined using multi-mode microplate reader (SpectraMax M5, Molecular Devices, Sunnyvale, CA).

### Methylated RNA immunoprecipitation (Me-RIP)

Total RNA was isolated from CRC cells by TRIzol method, and mRNA was isolated and purified from the total RNA using PolyATtract® mRNA Isolation Systems (A-Z5300, A&D Technology Corporation, Beijing, China). Antibody to M6A (1:500, ab151230, Abcam, Cambridge, UK) or antibody to IgG (ab109489, 1:100, Abcam, Cambridge, UK) was added to IP buffer (20 mM Tris pH 7.5, 140 mM NaCl, 1% NP-40, 2 mM EDTA) and incubated with protein A/G magnetic beads for 1 h for binding. Then purified mRNA and magnetic bead-antibody complexes were added to IP buffer with ribonuclease inhibitors and protease inhibitors, and incubated overnight at 4℃. Eluent buffer was used to elute RNA, which was then extracted and purified by phenol-chloroform. MYC in the extracted RNA was determined by RT-qPCR using the primer sequences depicted in Table 1.

### Photoactivatable ribonucleoside-enhanced crosslinking and immunoprecipitation (PAR-CLIP)

CRC cells were incubated with 200 mm of 4-thiopyridine (4SU) (Sigma Aldrich) for 14 h and cross-linked with 0.4J/cm2 at 365 nm. After lysis, immunoprecipitation was performed with FTO antibody (5 and 3 mg, respectively) at 4℃, after which the precipitated RNA was labeled with [g-32-p]-ATP, and observed by autoluminescence assay. Protein was removed by detachment using protease K, and RNA was extracted and subjected to RT-qPCR for MYC expression detection.

### Cell counting kit-8 (CCK-8) assay

CCK-8 assay kit (CK04, Dojindo, JPN) was utilized to analyze the viability of CRC cells. The cells at logarithmic growth phase, 1 × 10^4^ cells/well, were seeded into 96-well plates for 24 h of pre-culture, followed by 48 h transfection. At 0 h, 24 h, 48 h, 72 h post transfection, 10 µL of CCK-8 reagent was added to each well for reaction at 37℃ for 3 h. After that, absorbance at 450 nm was determined on a microplate reader, and a cell growth curve was drawn.

### Flow cytometric analysis

After transfection for 48 h, cells were collected and dispersed into cell suspension with 0.25% trypsin, 1 × 10^6^ cells/mL. As for cell cycle detection, 100 µL cell suspension was incubated with 50 µL PI dye containing RNAase avoiding light exposure for 30 min. Annexin V-fluorescein isothiocyanate (FITC)/propidium iodide (PI) staining was conducted for apoptosis assessment. In short, the cells were stained with 10 µL Annexin V-FITC (ab14085, Abcam, Inc., Cambridge) and 5 µL PI for 15 min devoid of light exposure. The cell cycle and apoptosis were assessed on a flow cytometer (BD Biosciences, FL, Lakes, NJ) at an exciting wavelength of 488 nm.

### Scratch test

Cells were seeded overnight into the 6-well plate at a density rate of 2.5 × 10^4^ cells/cm^2^. After scratches were made using a 200 µL pipette, the cells were cultured with DMEM containing 5% FBS. The images of scratches in each well at 0 and 24 h were captured under an inverted microscope. Three duplicates were set in each group. The width of each scratch was measured using the Image J software and the healing rate was calculated as follows: healing rate = (scratch width at 0 h - scratch width at 24 h)/scratch width at 0 h × 100%.

### Transwell assay

A total of 600 mL of DMEM containing 20% FBS was added to the lower chamber of Transwell chamber with polycarbonate membrane(8 µm pore) coated with Matrigel and incubated at 37℃ for 1 h. Cells following 48 h of transfection were resuspended in serum-free DMEM and seeded to the upper chamber at a density of 1 × 10^6^ cells/mL, followed by 24 h of incubation at 37℃ with 5% CO_2_. Then, Transwell chamber was removed and cells were washed twice with PBS and fixed with 5% glutaraldehyde. Afterwards, 0.1% crystal violet was added to the cells and stained for 5 min. After washing with PBS, the cells remaining on the upper surface were wiped away with a cotton swab. Finally, cells were observed under an inverted fluorescence microscope in five randomly selected visual fields, with mean values obtained.

### Nude mouse tumor xenograft model

Twelve specific-pathogen-free female BALB/c nude mice (aged 6 weeks; body weight of 15–18 g) were purchased from Shanghai SLAC Laboratory Animal co. LTD (Shanghai, China). CRC cells SW480 at logarithmic growth phase were prepared into cell suspension with a concentration of about 1 × 10^7^/100 µL, which was then injected into the left axilla of nude mice with a 1 ml syringe to establish a subcutaneous mouse xenograft model. Once the tumor volume reached about 50 mm^3^, the nude mice were injected with miR-96 antagomir or NC antagomir (10 nmol once every 5 days for 5 weeks). After 5 weeks, the mice were euthanized, after which the subcutaneous transplanted tumor was removed, and weighed. The protein was then extracted from the transplanted tumor tissues for Western blot analysis.

### Statistical analysis

SPSS 21.0 statistical software (IBM Corp. Armonk, NY) was utilized for data statistical analysis, with *p* < 0.05 as a level of statistically significance. Measurement data were expressed as mean ± standard deviation. Data between cancer tissues and paracancerous tissues were compared using paired *t*-test and data between other two groups were compared using unpaired *t*-test. Data among multiple groups were assessed by one-way analysis of variance (ANOVA), followed by Tukey’s post hoc tests for multiple comparisons. Time-based multi-comparison was tested by repeated measures ANOVA, with Bonferroni post hoc tests. Enumeration data were analyzed using chi-square test.

## Results

### miR-96 targeted AMPKα2 and inhibited its expression in CRC

**Fig. 1:**
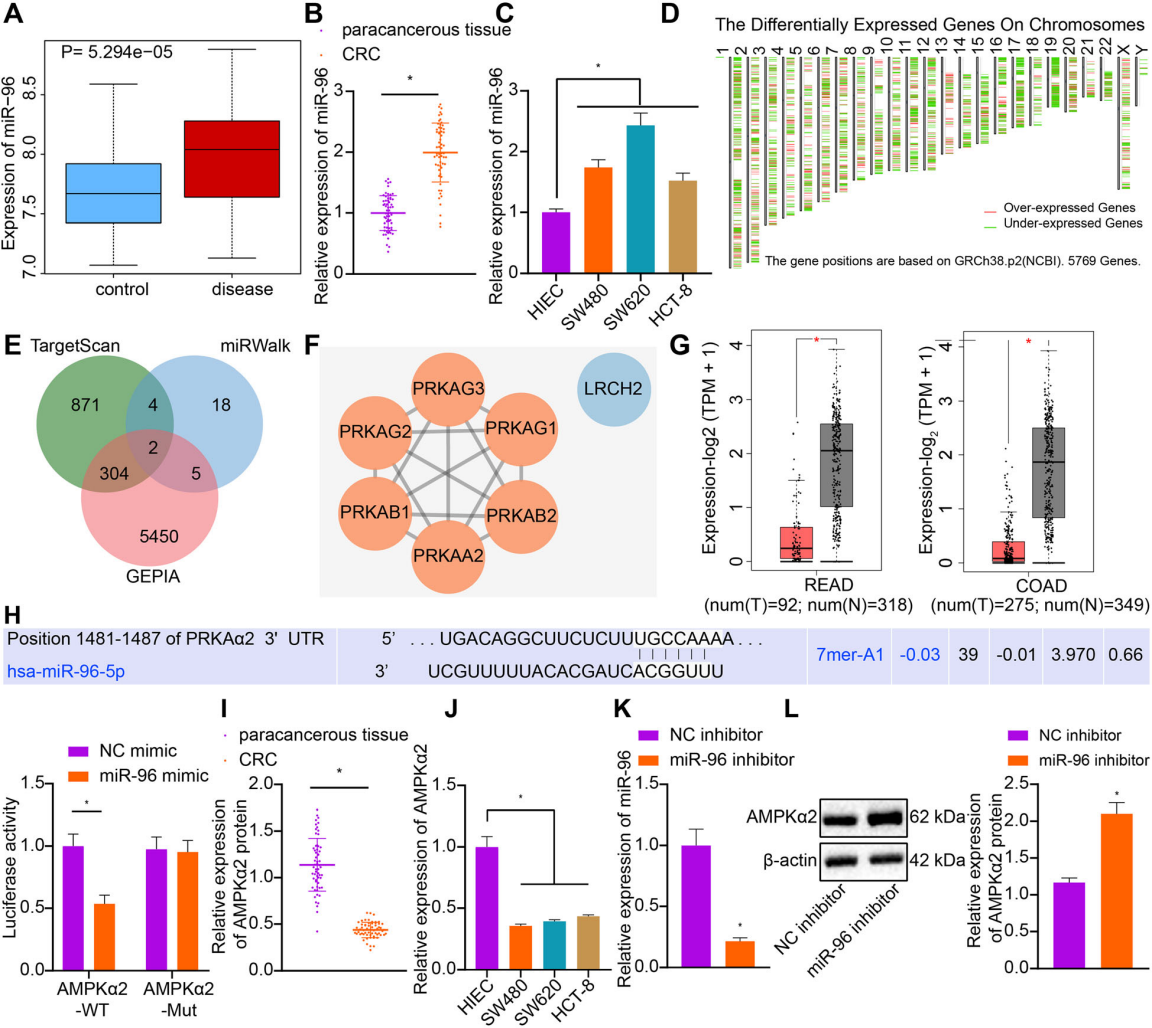
miR-96 targets and downregulates the expression of AMPKα2 in CRC. **a**: Boxplot of miR-96 expression in normal samples (left blue box) and CRC samples (right red box) obtained from microarray GSE38389 from the GEO database (https://www.ncbi.nlm.nih.gov/gds). **b**: The expression of miR-96 in clinical CRC tissues and non-tumor tissues determined by RT-qPCR (*n* = 60 for patients in each group). **c**: The expression of miR-96 in CRC cells and HIEC cells determined by RT-qPCR. **d**: Map of differentially expressed genes in TCGA database (https://portal.gdc.cancer.gov) analyzed by GEPIA (http://gepia2.cancer-pku.cn/#index). Red represents the significantly up-regulated genes, while green represents the significantly down-regulated genes. From left to right, chromosomes 1–22 and X and Y are in sequence. **e**: Venn diagrams of differentially expressed genes from GEPIA and downstream target genes of miR-96 from TargetScan (http://www.targetscan.org/vert_71/) and miRWalk (http://mirwalk.umm.uni-heidelberg.de); **f**: PPI network of genes related to LRCH2 and AMPKα2 constructed by String (https://string-db.org) and network image plotted by Cytoscape (https://cytoscape.org). The higher the core degree of the gene, the redder the circle, and conversely, the lower the core degree, the bluer the circle. **g**: The expression of AMPKα2 in CRC (left) and colon cancer (right) analyzed by GEPIA. The red box on the left represents that in cancer samples, while the gray box on the right represents that in normal samples. **h**: The binding of miR-96 to AMPKα2 predicted by TargetScan (http://www.targetscan.org/vert_71/) and confirmed by dual-luciferase reporter gene assay. **i**: The expression of AMPKα2 in clinical CRC tissues and non-tumor tissues determined by RT-qPCR (*n* = 60 for patients in each group). J: The mRNA expression of AMPKα2 in CRC cells and HIEC cells determined by RT-qPCR. K: The expression of miR-96 in CRC cells transfected with miR-96 inhibitor determined by RT-qPCR. L: The protein expression of AMPKα2 in CRC cells transfected with miR-96 inhibitor measured by Western blot analysis. Measurement data (mean ± standard deviation) between the two groups were analyzed by paired *t* test and those among multiple groups were compared by one-way ANOVA with Tukey’s post hoc tests. The cell experiment was independently repeated three times

miR-96 has been reported to have the ability to accelerate the occurrence of CRC [16], but the specific mechanism by which miR-96 controls the occurrence of CRC remains unclear. We first conducted microarray-based analysis and found that miR-96 was highly expressed in CRC in the GSE38389 dataset from the GEO database (Fig. 1a). Consistent with those findings, we experimentally determined that miR-96 expression was markedly increased in CRC tissues and cells as compared to the non-tumor tissues and HIEC cells by RT-qPCR (Fig. 1b and c). Importantly, 1181 and 29 downstream target genes of miR-96 were respectively retrieved from TargetScan and miRWalk databases, and 5761 differentially expressed genes were obtained by analyzing the data from TCGA database using GEPIA. LRCH2 and AMPKα2 (PRKAA2 in NCBI) were obtained through intersecting downstream target genes and differentially expressed genes (Fig. 1d-e). We next constructed the PPI network of LRCH2 and AMPKα2 using String, with the core degree calculated with Cytoscape. As depicted in Fig. 1f, the core degree of AMPKα2 was higher than that of LRCH2, and hence was the key downstream target gene of miR-96. The expression of AMPKα2 was found to be poorly expressed in CRC following GEPIA analysis (Fig. 1g). The TargetScan database revealed binding sites between miR-96 and AMPKα2 3’-UTR and dual-luciferase reporter assay verified the binding relationship between AMPKα2 and miR-96 (Fig. 1h). The luciferase activity of AMPKα2-WT was diminished in response to miR-96 mimic versus NC mimic, whereas that of AMPKα2-Mut exhibited no significant difference, indicating that miR-96 could specifically bind to AMPKα2. RT-qPCR assay (Fig. 1i and j) revealed that expression of AMPKα2 in CRC tissues and cells was notably diminished, as compared with the non-tumor tissues and HIEC cells. The subsequent loss-of-function experiments revealed that SW480 cells transfected with miR-96 inhibitor had diminished miR-96 expression (Fig. 1k) and increased AMPKα2 expression (Fig. 1l). These results indicated that miR-96, an upregulated miR in CRC, downregulated the expression of AMPKα2. Additionally, through correlation analysis, it was revealed that the expression of miR-96 and AMPKα2 was associated with tumor, node, metastasis classification and lymph node metastasis of CRC patients instead of the gender and age of patients (Table 2).

**Table 2:** Correlation analysis between the clinic opathological characteristics of CRC patients and the expression of miR-96 and AMPKα2

Clinic opathological characteristics	Case	miR-96			AMPKα2		
		High expression	Low expression	*P*	High expression	Low expression	*P*
Gender				0.4636			0.5731
Male	42	19	23		22	20	
Female	18	10	8		8	10	
Age (year)				0.2015			0.1205
< 60	32	13	19		19	13	
≥ 60	28	16	12		11	17	
TNM classification				< 0.001			< 0.001
I-II	28	4	24		23	5	
III-IV	32	25	7		7	25	
LNM				< 0.001			< 0.001
Yes	34	25	9		7	27	
No	26	4	22		23	3	

Note: The data were enumeration data and analyzed using chi-square test; *p* < 0.05 indicated that the difference was statistically significant. *CRC* colorectal cancer; *miR-96* microRNA-96; *TNM* tumor, node, metastasis; *LNM* lymph node metastasis

### miR-96 facilitated malignant phenotypes of CRC cells by targeting AMPKα2

**Fig. 2:**
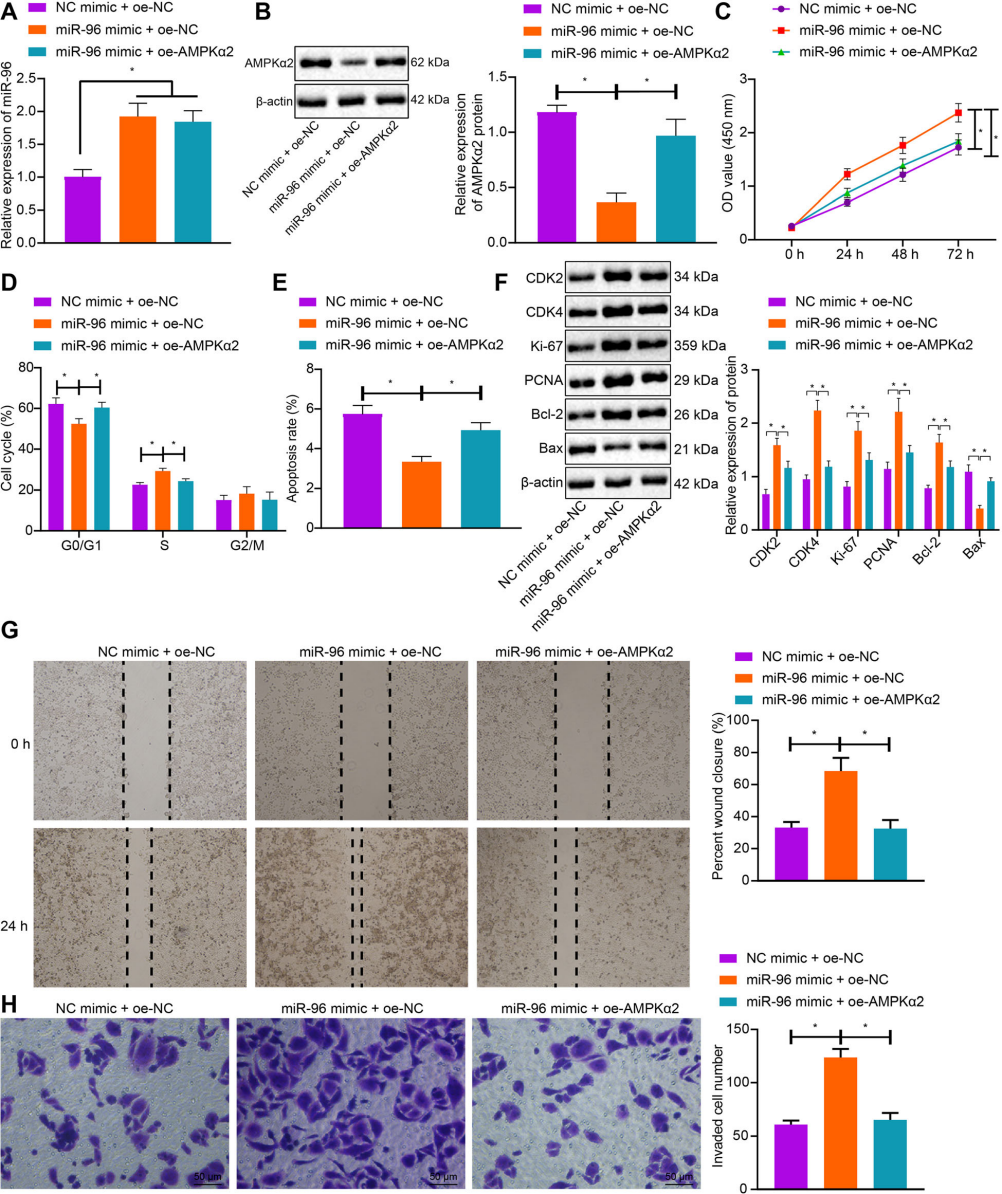
miR-96 enhances cell migration, proliferation and invasion while repressing apoptosis of CRC cells by inhibiting AMPKα2. **a**: The expression of miR-96 in CRC cells co-transfected with miR-96 mimic and oe-NC/oe-AMPKα2 determined by RT-qPCR. **b**: The protein expression of AMPKα2 in CRC cells co-transfected with miR-96 mimic and oe-NC/oe-AMPKα2 measured by Western blot analysis. **c**: Viability of CRC cells co-transfected with miR-96 mimic and oe-NC/oe-AMPKα2 assessed by CCK-8 method. **d**: Quantitative analysis for cell cycle distribution in CRC cells co-transfected with miR-96 mimic and oe-NC/oe-AMPKα2 detected by flow cytometry. **e**: Quantitative analysis for apoptosis rate of CRC cells co-transfected with miR-96 mimic and oe-NC/oe-AMPKα2 assessed by flow cytometry. **f**: The protein expression of proliferation-, cell cycle- and apoptosis-related genes in CRC cells co-transfected with miR-96 mimic and oe-NC/oe-AMPKα2, as measured by Western blot analysis; **g**: The migratory ability of CRC cells co-transfected with miR-96 mimic and oe-NC/oe-AMPKα2 assessed by scratch test. **h**, The invasion ability of CRC cells co-transfected with miR-96 mimic and oe-NC/oe-AMPKα2 assessed by Transwell assay. * *p* < 0.05. Measurement data (mean ± standard deviation) between the two groups were compared by paired *t* test and those among multiple groups at different time points were compared by repeated measures ANOVA with Bonferroni post-hoc test. The cell experiment was independently repeated three times

We next attempted to clarify the effect of miR-96 on the biological characteristics of CRC cells by targeting AMPKα2. As illustrated in Fig. 2a and b, the protein expression of AMPKα2 was reduced by miR-96 mimic, which was rescued by co-transfection with oe-AMPKα2. CCK-8 assay and flow cytometry validated that cell viability was markedly increased while cell cycle arrest and apoptosis were restricted by miR-96 mimic. On the contrary, restoration of AMPKα2 partially counteracted the pro-proliferative and anti-apoptotic effects of miR-96 (Fig. 2c-e). Moreover, Western blot analysis identified that protein expression of cell cycle-related proteins (CDK2 and CDK4), proliferation makers (Ki-67 and PCNA) and pro-apoptotic Bcl-2 were upregulated while that of anti-apoptotic Bax was diminished by miR-96 overexpression, whereas, all those changes induced by miR-96 mimic were reversed by co-transfection with oe-AMPKα2 (Fig. 2f). Besides, scratch test validated that cell migration was enhanced in response to miR-96 mimic, which was, whereas, reversed by the co-transfection with oe-AMPKα2 (Fig. 2g). Furthermore, analysis using Transwell assay revealed an enhancement of cell invasion following miR-96 overexpression, while further overexpression of AMPKα2 resulted in a decline (Fig. 2h). Together, miR-96 might stimulate cell proliferative, migratory and invasive capacities while inhibiting cell apoptotic potential by targeting AMPKα2.

### AMPKα2 impaired CRC cell proliferative, migratory and invasive capability, and induced apoptosis by suppressing FTO

**Fig. 3:**
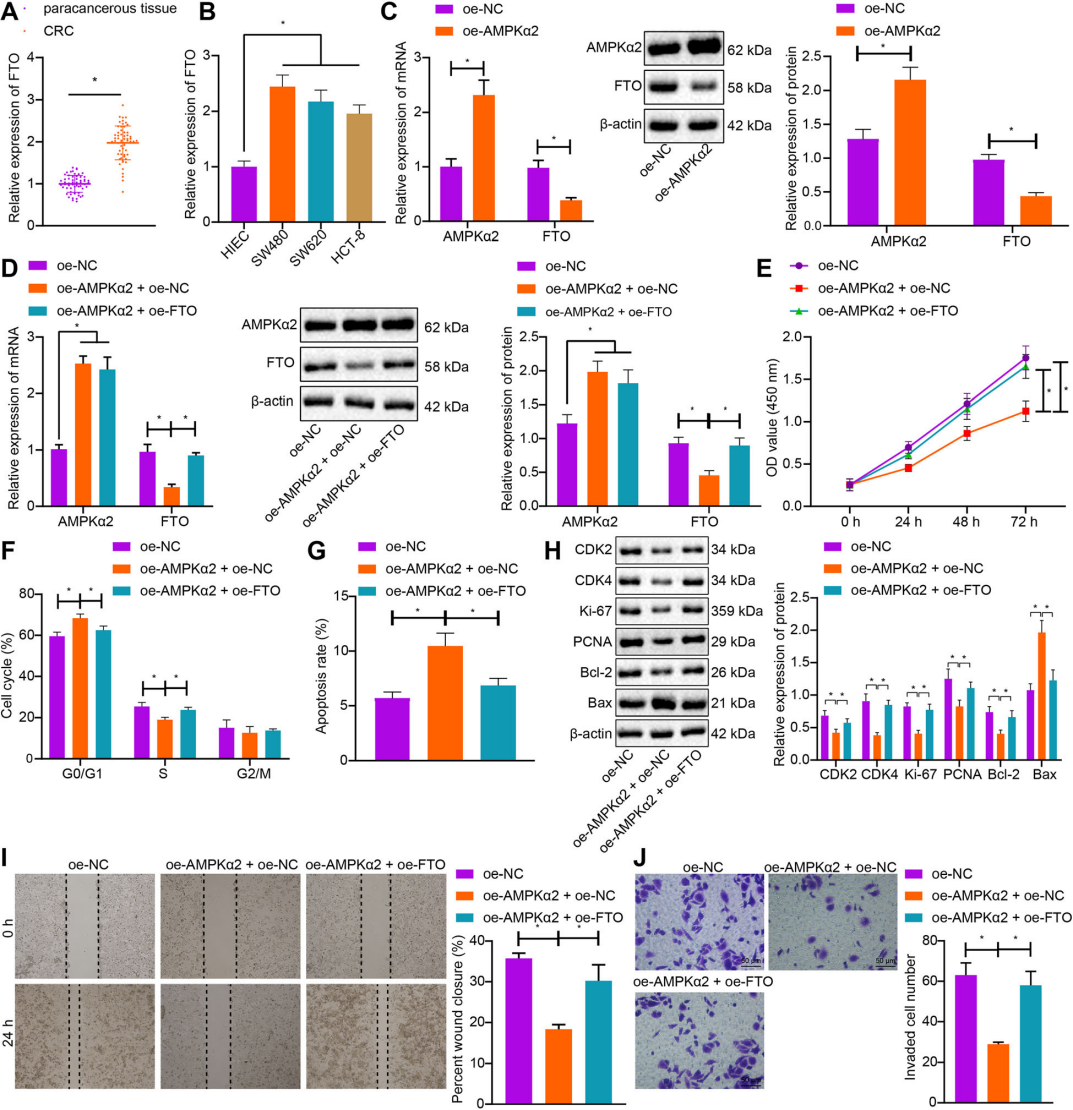
AMPKα2 inhibits CRC cell proliferation, migration, and invasion and induces apoptosis by inhibiting FTO. **a**: The mRNA expression of FTO in clinical CRC tissues and non-tumor tissues determined by RT-qPCR (*n* = 60 for patients in each group). **b**: The mRNA expression of FTO in CRC cells and HIEC cells determined by RT-qPCR. **c**: The protein expression of AMPKα2 and FTO in CRC cells transfected with oe-AMPKα2 measured by RT-qPCR and Western blot analysis. **d**: The protein expression of AMPKα2 and FTO in CRC cells co-transfected with oe-AMPKα2 and oe-NC/oe-FTO measured by RT-qPCR and Western blot analysis. **e**: Viability of CRC cells co-transfected with oe-AMPKα2 and oe-NC/oe-FTO assessed by CCK-8 method. **f**: Quantitative analysis for cell cycle distribution after co-transfection with oe-AMPKα2 and oe-NC/oe-FTO detected by flow cytometry. **g**: Quantitative analysis for apoptosis rate of CRC cells after co-transfection with oe-AMPKα2 and oe-NC/oe-FTO evaluated by flow cytometry. **h**: The expression of proliferation-, cell cycle- and apoptosis-related proteins in CRC cells co-transfected with oe-AMPKα2 and oe-NC/oe-FTO, as measured by Western blot analysis. **i**: The migratory ability of CRC cells co-transfected with oe-AMPKα2 and oe-NC/oe-FTO as detected by scratch test. **j**, The invasion ability of CRC cells co-transfected with oe-AMPKα2 and oe-NC/oe-FTO assessed by Transwell assay. ^*^p < 0.05. Measurement data (mean ± standard deviation) between the two groups were analyzed by paired *t* test and those among multiple groups were compared by one-way ANOVA with Tukey’s post hoc tests. Data among multiple groups at different time points were compared by repeated measures ANOVA with Bonferroni post-hoc test. The cell experiment was independently repeated three times

AMPKα2 has been found to impede the expression of FTO [19]. In this study, we first performed RT-qPCR and found an upregulation of FTO mRNA expression in CRC tissues and cells compared with the non-tumor tissues and HIEC cells (Fig. 3a and b). Next, the SW480 cells were transfected with oe-AMPKα2 or oe-NC or in combination with oe-FTO. AMPKα2 expression was elevated in the SW480 cells transfected with oe-AMPKα2, while that of FTO was notably diminished, as illustrated by RT-qPCR and Western blot analysis (Fig. 3c). Besides, the oe-AMPKα2-inhibited FTO expression was restored by co-transfection with oe-FTO (Fig. 3d). These results suggested that AMPKα2 downregulated FTO at protein and mRNA levels in CRC cells. Subsequently, we aimed to investigate the effect of AMPKα2 on the biological characteristics of CRC cells by inhibiting FTO. Diminished viability of SW480 cells, more cells arrested at G0/G1 phase and fewer cells arrested at S phase in addition to enhanced cell apoptosis were observed upon overexpression of AMPKα2 (Fig. 3e-g). However, those effects of AMPKα2 on SW480 cells were negated by additional transfection of oe-FTO. Additional data obtained from immunoblotting displayed a reduction in the protein expression of CDK2, CDK4, Ki-67, PCNA and Bcl-2 and an elevation of Bax protein expression upon upregulation of AMPKα2. By contrast, reversed changes were observed regarding the aforementioned factors when SW480 cells underwent co-transfection with oe-AMPKα2 and oe-FTO (Fig. 3h). As shown by scratch test, cell migratory ability was repressed by the transfection with oe-AMPKα2 but potentiated by the co-transfection with oe-AMPKα2 and oe-FTO (Fig. 3i). Furthermore, analysis using Transwell assay revealed a decline of cell invasion following AMPKα2 overexpression, while further overexpression of FTO resulted in opposite results (Fig. 3j). Collectively, AMPKα2 could augment cell apoptosis and impair proliferative, migratory and invasive capacities *via* downregulation of FTO.

### FTO augmented the expression of MYC by removing the m6A modification of MYC in CRC cells

**Fig. 4:**
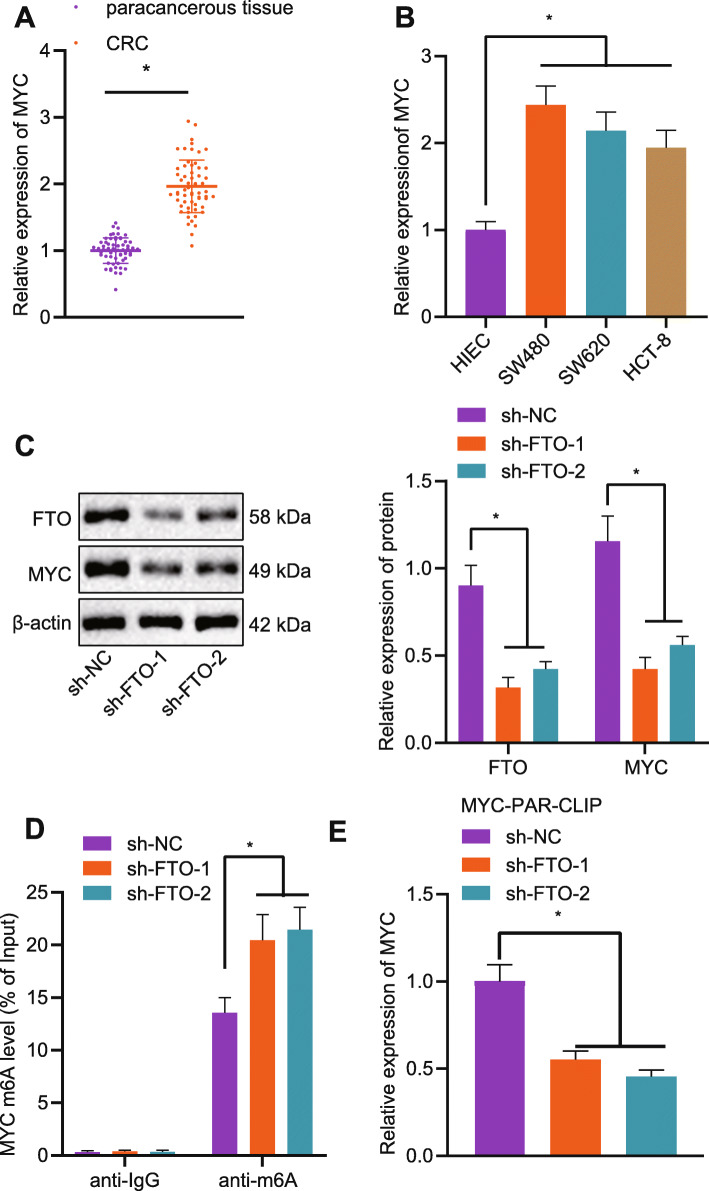
Demethylase FTO elevates the expression of MYC by blocking the m6A modification of MYC in CRC cells. **a**: The mRNA expression of MYC in clinical CRC tissues and non-tumor tissues determined by RT-qPCR (*n* = 60 for patients in each group). **b**: The mRNA expression of MYC in CRC cells and HIEC determined by RT-qPCR. **c**: The protein expression of FTO and MYC in CRC cells after transfection with sh-FTO measured by Western blot analysis. **d**: The m6A modification level of MYC in CRC cells assessed by Me-RIP assay. **e**: The binding relationship between FTO and MYC mRNA examined by PAR-CLIP assay; ^*^p < 0.05. Measurement data (mean ± standard deviation) between the two groups were compared by paired *t* test and those among multiple groups were compared by one-way ANOVA with Tukey’s post hoc tests. Cell experiment was independently repeated for three times

FTO has been noted to interact with MYC involving in the CRC cell migration [18]. The RT-qPCR in CRC clinical tissues and cells identified highly expressed MYC expression, as depicted in Fig. 4a and b. After successful FTO knockdown by transfection with sh-FTO, MYC protein expression was reduced (Fig. 4c). In addition, FTO knockdown resulted in enhanced m6A modification level (Fig. 4d). As shown in Fig. 4e, PAR-CLIP assay revealed that the pulled-down MYC mRNA expression was noticeably reduced upon FTO silencing. The above results together revealed that the demethylase FTO elevates MYC expression by impairing the m6A modification of the MYC gene in CRC.

### FTO induced CRC cell proliferative and invasive capacities and hindered apoptotic ability through upregulation of MYC

**Fig. 5:**
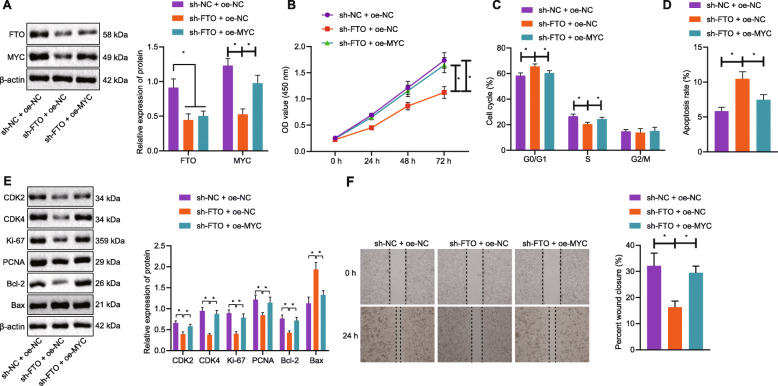
Demethylase FTO restrains CRC cell proliferation, migration, cell cycle progression and facilitates cell apoptosis through upregulation of MYC. **a**: The protein expression of FTO and MYC in CRC cells co-transfected with sh-FTO/sh-NC and oe-NC/oe-MYC measured by Western blot analysis. **b**: The viability of CRC cells co-transfected with sh-FTO/sh-NC and oe-NC/oe-MYC assessed by CCK-8 method. **c**: Quantitative analysis for cell cycle distribution in CRC cells co-transfected with sh-FTO/sh-NC and oe-NC/oe-MYC assessed by flow cytometry. **d**: Quantitative analysis for apoptosis rate of CRC cells co-transfected with sh-FTO/sh-NC and oe-NC/oe-MYC evaluated by flow cytometry. **e**: The expression of proliferation-, cell cycle- and apoptosis-related proteins in CRC cells co-transfected with sh-FTO/sh-NC and oe-NC/oe-MYC, as measured by Western blot analysis. **f**: The migratory ability of CRC cells co-transfected with sh-FTO/sh-NC and oe-NC/oe-MYC, as measured using scratch test. ^*^p < 0.05. Measurement data (mean ± standard deviation) among multiple groups were compared by one-way ANOVA with Tukey’s post hoc tests. Repeated measures ANOVA was used for data comparison between groups at different time points, followed by Bonferroni post hoc test. Cell experiment was independently repeated three times

In the following experiment, we set out to explain the modulatory effect of FTO-mediated upregulation of MYC on the biological characteristics of CRC cells. MYC protein expression was expectably reduced when FTO was silenced in CRC cells (Fig. 5a). By contrast, MYC protein expression was rescued by co-transfection with oe-MYC in the absence of FTO. As displayed in Fig. 5b-d, silencing of FTO resulted in reduction in cell viability and the number of cells arrested at S phase in addition to enhancement of apoptotic ability. However, oe-MYC contributed to neutralizing the effects of sh-FTO on those abilities of CRC cells. Subsequent results of Western blot analysis displayed that FTO loss-of-function led to a reduction in expression of CDK2, CDK4, Ki-67, PCNA and Bcl-2 proteins but an increase in that of Bax protein. By contrast, oe-MYC transfection reversed the changes in above-mentioned proteins induced by sh-FTO (Fig. 5e). Moreover, scratch test showed that sh-FTO transfection resulted in restricted migration while the co-transfection with oe-MYC in the absence of FTO led to elevated migratory ability (Fig. 5f). Hence, FTO was suggested to stimulate CRC cell proliferative and invasive capacities and repress apoptotic ability *via* enhancement of MYC.

### miR-96 mediates m6A modification of MYC by regulating AMPKα2

**Fig. 6:**
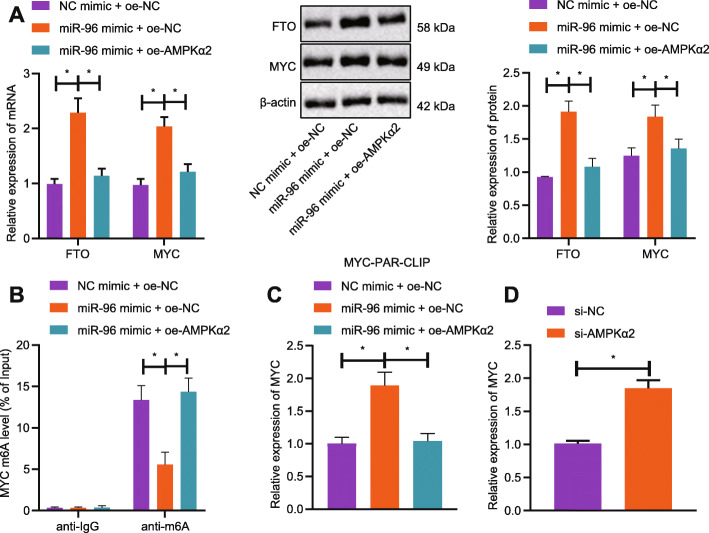
miR-96 impairs m6A modification of MYC by targeting AMPKα2. **a**: The protein expression of FTO and MYC in CRC cells co-transfected with miR-96 mimic/NC mimic and oe-NC/oe-AMPKα2 measured by Western blot analysis. **b**: The m6A modification level of MYC in CRC cells co-transfected with miR-96 mimic/NC mimic and oe-NC/oe-AMPKα2 assessed by Me-RIP. **c**: The binding relationship between FTO and MYC mRNA examined by PAR-CLIP assay; **d**: Expression of MYC following the transfection of si-AMPKα2 detected by RT-qPCR. ^*^p < 0.05. Measurement data (mean ± standard deviation) among multiple groups were compared by one-way ANOVA with Tukey’s post hoc tests. The cell experiment was independently repeated for three times

To probe into whether miR-96 indirectly controls the m6A modification of MYC, the expression pattern of FTO and MYC in response to miR-96 gain-of-function. As shown by RT-qPCR and western blot analysis, the mRNA and protein expression of both FTO and MYC was elevated when the CRC cells underwent co-transfection with miR-96 mimic and oe-NC while it was diminished upon co-transfection with miR-96 mimic and oe-AMPKα2 (Fig. 6a). Meanwhile, Me-RIP assay exhibited that miR-96 upregulation resulted in a reduction in m6A modification level of MYC but this reduction was diminished by oe-AMPKα2 (Fig. 6b). As shown by PAR-CLIP assay, the expression of the pulled-down MYC mRNA was elevated when miR-96 was upregulated, which was reversed by co-treatment of miR-96 mimic and oe-AMPKα2 (Fig. 6c). When compared with the transfection with si-NC, transfection with si-AMPKα2 led to elevated MYC expression (Fig. 6d).

### **Downregulation of miR-96 restricted the growth of CRC transplanted tumors*in vivo***

**Fig. 7:**
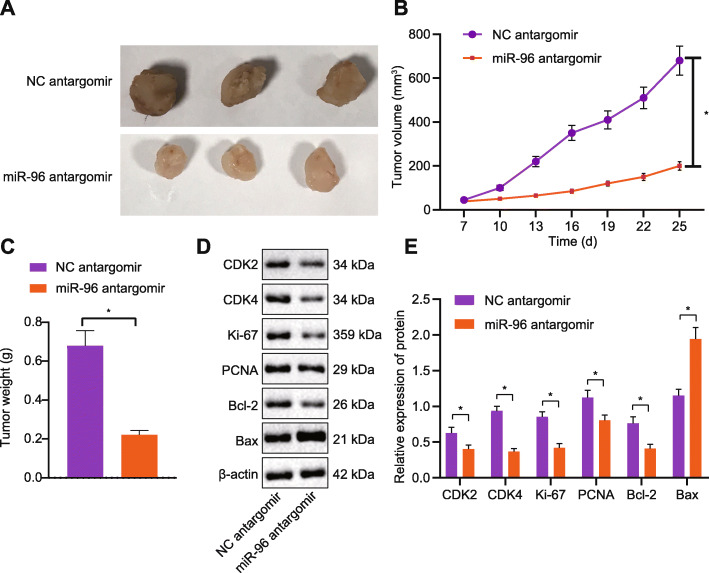
miR-96 inhibits the growth of CRC *in vivo*. **a**-**c**: Representative images of xenograft tumors (**a**) and quantitative analysis of tumor volume (**b**) and weight (**c**) after administration of miR-96 antagomir; **d**-**e**: Protein expression of FTO, MYC, CDK2, CDK4, Ki-67, PCNA, Bcl-2, AMPKα2 and Bax in tumor tissues of mice after administration of miR-96 antagomir measured by Western blot analysis. ^*^p < 0.05. Measurement data (mean ± standard deviation) between the two groups were compared by unpaired *t* test and those among multiple groups at different time points were compared by repeated measures ANOVA with Bonferroni post hoc test. *n* = 6

For the purpose of assessing the effect of miR-96 on the growth of CRC transplanted tumors *in vivo*, we developed xenograft tumor model in nude mice by injection with CRC cell suspension. The miR-96 antagomir was administrated into tumor-bearing mice to knockdown miR-96 expression. Consistent with the *in vitro* findings, tumor volume and weight in the presence of miR-96 antagomir were significantly reduced (Fig. 7a-c). Proteins from transplanted tumor tissues were extracted for Western blot analysis, and the results displayed that levels of FTO, MYC, CDK2, CDK4, Ki-67, PCNA and Bcl-2 proteins in miR-96 antagomir group were downregulated, while AMPKα2 and Bax proteins were raised (Figs. 4e and 7d). The anti-tumor role of miR-96 antagomir was therefore confirmed.

## Discussion

A minority of the CRC population are affected by the genetic mutations of oncogenes, anti-oncogenes or miRs [17–19]. Microarray profiling has identified a large range of miRNAs dysregulated in human CRC tissues, in contrast to adjacent non-cancerous tissues [20, 21]. Herein, our study intended to elucidate the functions and mediatory mechanisms of miR-96 in CRC.

In our study, we found miR-96 could augment CRC cell proliferative and invasive capacities, and suppress apoptotic ability by targeting AMPKα2. Consistent with our finding, miR-96 has been documented to be highly expressed in CRC tissues versus normal mucosal tissues [21], and this upregulation in CRC has also been confirmed in other researchers [22–24]. In addition, miR-96 has been clinically validated to be a circulating biomarker for predicting the overall survival of CRC patients [25]. Serum miR-96 is also proposed to be an indicator distinguishing chemoresistance in the advanced CRC [26]. This value may be correlated with the role of miR-96 [27]. More recently, miR-96 inhibitor could function as a suppressor of cell migration instead of cell invasion that may be affected by the counteraction of cell invasion stimulator Matrigel [13]. In the present study, we validated the anti-tumor activity exerted by miR-96 antagomir in the nude mouse model.

Emerging evidence demonstrates that miRNAs participate in controlling cancer cell growth, invasiveness and metastatic potential through interacting with the 3’UTR of specific target mRNAs [28, 29]. In a previous study, miR-96 has been substantiated to contribute to colorectal carcinogenesis *via* targeting TP53INP1, FOXO1 and FOXO3a [30]. AMPKα2 was identified to be a key target of miR-96 and restoration of AMPKα2 could partially reverse the pro-proliferation and anti-apoptosis functions of miR-96. This suggested that miR-96 exerted oncogenic role *via* targeting AMPKα2. AMPK is a hub sensor for cellular energy and nutrition. Ablation of AMPK or its deregulation has been observed in cancer and may interact with oncogenic drivers to mediate tumor cell metabolism [31]. Restored expression of AMPKα2 has been associated with the tumor attenuation in human breast and bladder cancers [32–34].

Further, AMPKα2 was suggested in this study to impede CRC cell proliferative and invasive capacities, while inducing apoptotic ability by repressing FTO. AMPKα2 has been formerly evidenced to repress the expression of FTO [16]. It was previously demonstrated that FTO could serve as a target gene of miR-1266 and was negatively modulated by miR-1266 in CRC [35]. MYC, whose deregulation has been found in most cancers including CRC, has a pivotal role to play in the tumorigenesis and carcinogenesis of CRC via the Wnt/β-catenin pathway [36]. In CRC cells, FTO elevates the expression of MYC by blocking the modification of MYC gene m6A. Consistently, this mechanism has been proposed in another study that FTO mediates m6A modification to accelerate the expression of MYC [37]. Tang et al. have suggested the interplay between FTO and MYC responsible for the pancreatic cancer cell proliferation [38]. A recent study demonstrated that FTO could cooperate with MYC for the enhancement of the proliferative and migrative functions of CRC cells [15]. On the basis of the aforementioned findings demonstrated by our study, we reasoned that miR-96 contributed to tumorigenesis *via* the AMPKα2/FTO/m6A/MYC axis.

## Conclusion

In summary, miR-96 could potentially stimulate malignancy and aggressiveness of CRC by activating AMPKα2-mediated FTO/MYC. The *in vivo* mouse model corroborated the anti-tumor role of miR-96 antagomir, highlighting that ablation of miR-96 could serve as a therapeutic target for CRC treatment. Further studies with larger cohorts are required to verify these findings and develop the translational values of this investigation.

## Data Availability

The datasets generated and/or analyzed during the current study are available from the corresponding author on reasonable request.

## Abbreviations

CRC: Colorectal cancer
miRNAs or miRs: microRNAs
m6A: N6-methyladenosine
FTO: Alpha-ketoglutarate-dependent dioxygenase
NC: negative control
oe-NC: overexpression plasmid
oe-AMPKα2: AMPKα2 overexpression plasmid
oe-FTO: FTO overexpression plasmid
sh-NC: NC shRNA
sh-FTO: shRNA targeting FTO
oe-MYC: MYC overexpression plasmid
RT-qPCR: Reverse transcription quantitative polymerase chain reaction
HEK: Human embryo kidney
Me-RIP: Methylated RNA immunoprecipitation
PAR-CLIP: Photoactivatable ribonucleoside-enhanced crosslinking and immunoprecipitation
4SU: 4-thiopyridine
ANOVA: analysis of variance
FBS: fetal bovine serum
DMEM: Dulbecco's modified Eagle's medium
FITC: fluorescein isothiocyanate
PI: propidium iodide
